# RISE: a novel unified framework for feature relevance in malnutrition analytics integrating statistical and expert insights

**DOI:** 10.3389/fpubh.2025.1663373

**Published:** 2025-10-16

**Authors:** S. Shruthi, Priya Govindarajan, S. R. Shalini, Pavan John Antony, A. N. Uma, Lalith Rangarajan

**Affiliations:** ^1^Department of Computer Science, School of Computing, Amrita Vishwa Vidyapeetham, Mysuru, India; ^2^Pediatric Department, Mysore Medical College and Research Institute, Mysuru, India; ^3^School of Education, Adelphi University, Garden City, NY, United States; ^4^Genetic Unit, Anatomy Department, Mahatma Gandhi Medical College and RI, Sri Balaji Vidyapeeth (Deemed to be University), Puducherry, India; ^5^Department of Studies in Computer Science, University of Mysore, Mysuru, India

**Keywords:** child malnutrition, maternal malnutrition, feature scoring, model-based scoring, domain-based scoring, filter-based feature Scoring, frequency boosting, XGBoost

## Abstract

Addressing child malnutrition remains a critical global health priority, directly contributing to Sustainable Development Goals (SDG 2 – Zero Hunger and SDG 3 – Good Health and Well-being). This study aims to identify and prioritize the most influential determinants of acute forms of malnutrition among children aged 0–23 months by developing a novel feature scoring framework, RISE (Relevance-based Integration of Statistics and Expertise). The objective is to bridge the gap between data-driven modeling and context-specific insights by integrating model-based scores (from XGBoost), statistical filter methods for frequency boosting, and domain-informed adjustments. Using real-world data from Nutrition Rehabilitation Centre (NRC) at K.R. District Hospital, Mysuru, the RISE framework enhances the interpretability and contextual relevance of predictors often underweighted in traditional models. Domain-relevant features such as Mother Height, Breastfeeding Status, Caste, Maternal Working Status, and Ration card emerged as critical factors when adjusted through the RISE Framework. The top-ranked features included Child Weight, maternal anthropometry, and Child order remained consistently influential determinants, reflecting maternal dependency and the double burden of malnutrition. RISE uncovers hidden yet meaningful contributors that often go underrepresented in purely model-driven analyses. By adjusting feature scores to recognize both empirical strength and domain importance. By aligning analytical rigor with public health relevance, this study contributes a scalable, context-sensitive approach to feature prioritization in malnutrition research, supporting more informed, targeted interventions and policy actions toward achieving global nutrition goals.

## 1 Introduction

Malnutrition remains one of the most persistent global health challenges, with every country striving to control, manage, and ultimately eradicate it. Malnutrition of any form, such as wasting, stunting, or underweight (1), can occur at various stages of life; its occurrence during childhood is considered the most alarming. Globally, stunting affected an estimated 23.2 percent or 150.2 million children, and wasting threatened the lives of an estimated 6.6 percent or 42.8 million children under 5 in 2024 (2). The outcome of the “Fifth National Family Health Survey (NFHS-5)” discloses a prevalence of malnutrition in India, children under 5 years who are “stunted (height-for-age) 35.5, ‘wasted (weight-for-height)’ 19.3, ‘severely wasted (weight-for-height)’ 7.7, ‘underweight (weight-for-age)’ by the ‘World Health Organization’ (WHO) standards (3)”. Both national governments and international organizations have recognized its critical impact and continue introducing policies, programs, and guidelines to address this multifaceted issue. Addressing malnutrition during the formative years is a healthcare imperative and a socioeconomic priority. Malnutrition is influenced by region-specific, environmental, cultural, and socioeconomic factors (2–5). What contributes to malnutrition in one setting may not hold the same relevance in another, making context-sensitive analysis crucial (5). Policymakers prioritize the identification of malnutrition determinants before formulating or revising any policies or guidelines (4). Understanding these underlying factors is essential for effective intervention design, resource allocation, and impact measurement. As a result, the study of determinants of malnutrition has emerged as a critical area of research.

In recent years, machine learning (ML) has emerged as a powerful tool for identifying malnutrition determinants. Conventional feature selection methods, such as statistically based filter methods, have been widely used in ML to identify relevant features by evaluating statistical relationships between features and the outcome. These techniques are computationally efficient and easy to interpret, making them valuable tools in the early stages of exploratory data analysis (6–9). However, they often evaluate features independently, ignoring potential interactions among variables. These methods are sensitive to data distribution, scale, and noise, and may overlook contextually important features that do not exhibit strong statistical signals. This limitation becomes critical in complex public health problems like malnutrition, where domain knowledge and latent factors play a significant role. Hence, relying solely on conventional techniques may lead to the exclusion of key features that are crucial from a policy or intervention perspective (10–14). The research objective is to develop and implement the RISE (relevance-based integration of statistics and expertise) framework to enhance the identification of key determinants of acute forms of child malnutrition. By integrating model-based feature importance, domain knowledge, and frequency boost based on statistical relevance, the study aims to capture both dominant and overlooked factors that traditional statistical and machine learning models overlook. The RISE balances predictive power with contextual significance. Computationally, it introduces frequency boosting using a filter-based ensemble model, normalization across scales, and implementation of nested grid search for hyperparameter tuning. These positions RISE as both a technically rigorous and domain-sensitive framework, filling a critical gap in malnutrition-related ML research.

### 1.1 Literature review

The application of machine learning (ML) to malnutrition prediction has evolved significantly over the past few years. Early work by Anku et al. (7) demonstrated the superiority of XGBoost (98% accuracy, 100% AUC) in predicting wasting, stunting, and underweight in Ghana. Talukder et al. (8) demonstrated the potential of ML algorithms such as random forest (RF), logistic regression, and k-nearest neighbors (k-NN) in identifying malnutrition among children, with RF achieving the highest sensitivity (94.66%) and specificity (69.76%). In 2021, Fenta et al. (9) conducted a comparative evaluation of six ML models—including logistic regression, LASSO, ridge regression, elastic net, neural networks, and RF across Ethiopian zones and established RF as the top-performing algorithm based on sensitivity, specificity, accuracy, and AUC. That same year, Bitew et al. (10) employed five ML methods, including XGBoost, RF, neural networks, and k-NN to predict socio-demographic risk factors of undernutrition, with XGBoost showing the highest accuracy (88.0%). Khan et al. (11) further validated the utility of ensemble methods by identifying gradient boosting as the most accurate model for predicting stunting among children under five years old. Additionally, Vasu et al. (12) employed the Boruta algorithm in conjunction with RF and PCA for dimensionality reduction to identify the most impactful features in malnutrition prediction. In 2022, Mohammad et al. (13) proposed an optimized hybrid approach combining Harris Hawk Optimization (HHO) with ADASYN for defect prediction, achieving classification accuracies exceeding 99%, with clear implications for imbalanced malnutrition datasets. In 2023, Ndagijimana et al. (14) used ensemble methods including gradient boosting and RF in Rwanda, where gradient boosting achieved the best performance (AUC 89%). The year 2024 has seen even broader adoption: Turjo et al. (15) used six classifiers, including RF and gradient boosting, to assess women’s malnutrition in Bangladesh, where RF showed the highest accuracy and AUC (0.604), Mkungudza et al. (16) applied seven variable selection techniques to logistic regression models to predict undernutrition with modest AUC performance (64%), Boruta feature selection, and the variables’ importance scores were used to identify determinants of malnutrition (17). Yalçın et al. (18) applied elastic net and RF models in neonatal intensive care settings, showing the models’ capacity to accelerate early malnutrition risk identification. This meta-analysis indicated that ML models were observed to have moderate to good performance metrics in predicting malnutrition using DHS data among children under five years (19). Collectively, studies reflect a growing confidence in machine learning models such as SVM, k-NN and ensemble methods, particularly RF. Gradient Boost and XGBoost, for accurate and scalable malnutrition prediction (20–23), though they also reveal ongoing challenges in feature selection, interpretability, and standardization of methodologies.

### 1.2 Research gap

Machine learning techniques have significantly advanced the identification of malnutrition determinants; they are not without limitations, particularly in how they handle feature importance and inherent model bias. One critical challenge is that statistical variance, feature distribution, and model architecture heavily influence most model-driven feature importance scores (7, 8). As a result, features that may hold substantial contextual or domain relevance can be assigned low importance because they exhibit weak correlations or appear less frequently in the training data. Furthermore, tree-based models like Random Forests or boosting algorithms may exhibit bias towards features with more unique values or features that dominate the data (9–12). This can lead to the underrepresentation of subtle but crucial socio-cultural or environmental factors. When models are trained on imbalanced or non-representative datasets, they may generalize poorly across regions or demographic groups, further skewing the feature rankings. While multiple studies apply ML algorithms, there is no uniform approach to feature selection, preprocessing, or handling of imbalanced data. Techniques like Boruta, PCA, and ADASYN are used in isolation (12–18), without comparative evaluations or integration into a common pipeline. Most models rely on statistical or wrapper-based feature selection methods (e.g., LASSO, Boruta), but very few studies integrate public health expertise or contextual domain relevance into feature importance ranking (19–21). This leads to the potential exclusion of sociocultural significant predictors that are weakly correlated in raw data but crucial in practice. These challenges make it difficult for policymakers to rely solely on raw model outputs, highlighting the need for more balanced frameworks that integrate statistical learning with human expertise to capture both machine-relevant and domain-important features. To address the limitations of traditional machine learning models in identifying determinants of malnutrition (22, 23), this study proposes the RISE (Relevance-based Integration of Statistics and Expertise) framework. RISE integrates statistical scores, model-based importance, and domain knowledge to uncover both dominant and overlooked features influencing acute forms of child malnutrition.

## 2 Methods

### 2.1 Study design, setting, and population

A cross-sectional study was conducted at Mysuru Medical College and Research Center, in collaboration with the Nutrition Rehabilitation Centres (NRC), from March 2024 to January 2025. The sample included 208 children aged 1–23 months admitted to the NRC, and children included in the study were identified with Moderate Acute Malnutrition (MAM) and Severe Acute Malnutrition (SAM), with data on maternal and child anthropometry, and socio–demographic information extracted from hospital records.

### 2.2 Characteristics of the study population

The study population included child, maternal, and socio–demographic information. The child’s information includes 56.8% males and 43.2% females. Children were categorized into four age groups: Group 1 (0–5 months) had 16.0% children, Group 2 (6–11 months) had 40.8% children, Group 3 (12–17 months) included 27.7% children, and Group 4 (18–23 months) comprised 15.5% children. Regarding birth weight, 60.7% children have normal birth weight. Anthropometric measurements showed that the mean Mid-Upper Arm Circumference (MUAC) was 11.57 cm (± 2.10 cm), the average weight was 6.32 kg (± 1.25 kg), and the mean height was 69.51 cm (± 6.71 cm), Among the 206 children included in the study, 51.5% were identified MAM and 48.5% with SAM, reflecting considerable variability in physical growth indicators across the sample. During admission to the NRC, child feeding practices were recorded. The data revealed overlapping patterns; 88.35% of children received breastfeeding, and 73.79% received bottle feeding. Specifically, 66.02% of children were fed both, 22.33% were exclusively breastfed, 7.77% were exclusively bottle-fed, and 3.88% received neither. These overlaps reflect concurrent feeding practices reported by caregivers and were retained to preserve the full behavioral context in the analysis.

Under maternal characteristics, the majority of mothers, 56.31% in the study were between 20–25 years. 52.91% of the mothers were classified as having an inadequate BMI, highlighting a significant burden of undernutrition among caregivers. Educational attainment with 65.05% having secondary education, though 4.85% remained illiterate. Regarding employment status, a substantial 91.26% were housewives, reflecting low workforce participation. In terms of reproductive profile, 60.19% of mothers had two or more children, and 56.31% of index children were of second or higher birth order, suggesting high family responsibility. 72.82% of mothers had not adopted any family planning methods, indicating a gap in reproductive health awareness and access.

**Figure 1:**
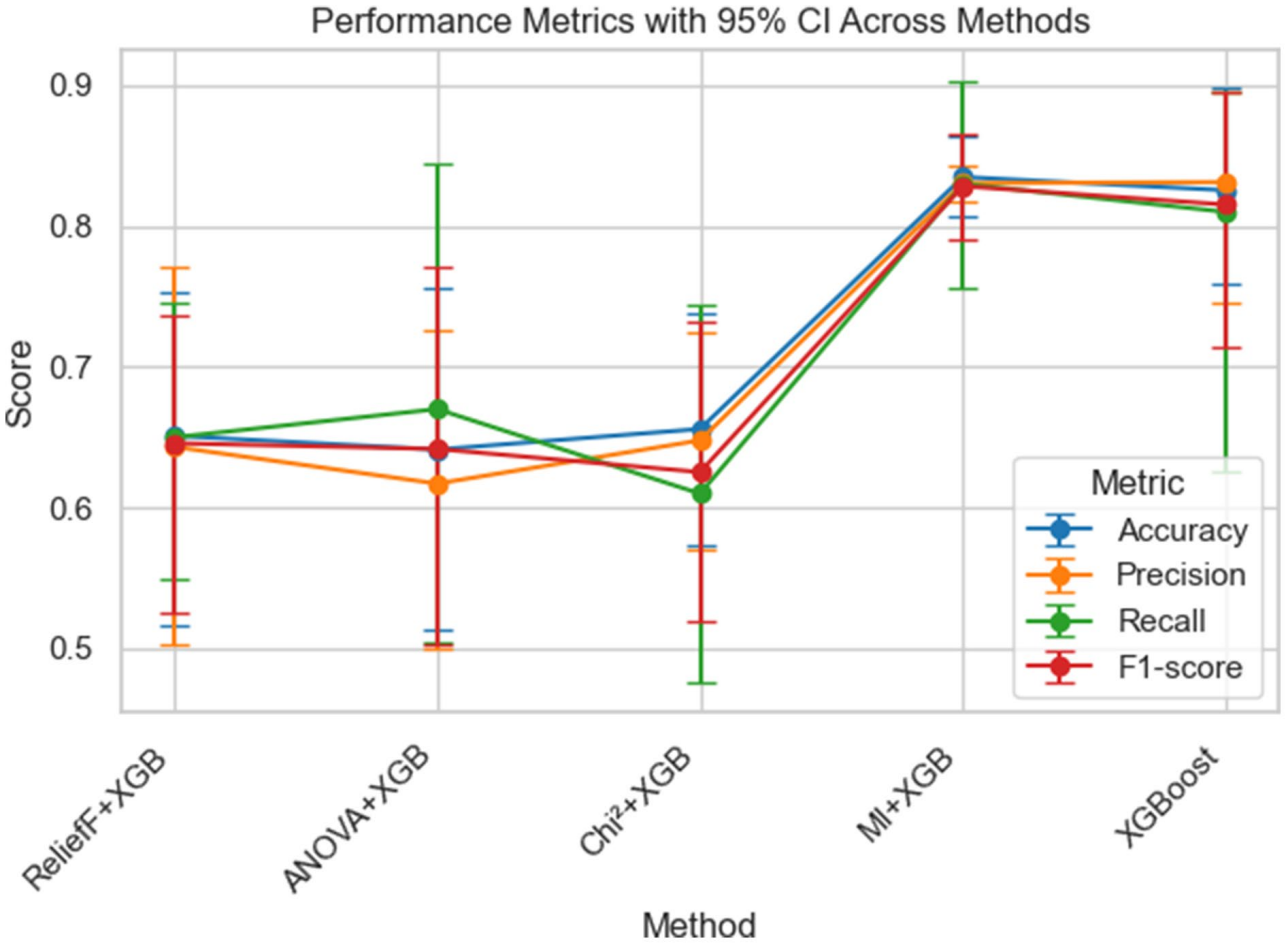
Comparative unified performance metrics with 95% confidence intervals across filter-based feature selection methods combined with XGBoost. Comparison of performance metrics with 95% confidence intervals across different filter-based feature selection methods combined with XGBoost. The plot shows Accuracy, Precision, Recall, and F1-score for five methods: ReliefF + XGB, ANOVA + XGB, Chi^2^ + XGB, MI + XGB, and XGBoost. Bars represent the 95% confidence intervals for each metric.

The socio-demographic analysis reveals that 87.86% families reported a non-vegetarian diet. 50.97% of the mothers belonged to Other Backward Classes (OBC), followed by 36.41% from Scheduled Castes and Scheduled Tribes (SC/ST), and 12.62% from the General category. 66.5% of the children were from rural areas. The Ration Card feature in the dataset comprises two categories: Above Poverty Line (APL) and Below Poverty Line (BPL), with BPL accounting for approximately 99.51% of the records. Given this near-constant distribution, a sensitivity analysis was conducted by executing the model both with and without this feature. The results showed no change in model performance metrics or feature rankings. For reference, the model maintained an overall accuracy of 82.54%, as shown in Figure 1, indicating that the inclusion of this low-variance variable did not influence predictive accuracy. This reinforces the robustness of the model and confirms that its outputs are not dependent on statistically redundant features.

### 2.3 Variables, definitions, and encoding

WHO Anthro software was used to compute Height for Age (HAZ), Weight for Age (WAZ), and Weight for Height (WHZ) scores based on each child’s age, height, and weight from NRC records. Nutritional status was classified as SAM (WHZ < −3 SD) and MAM (WHZ between −3 and −2 SD) (24).

Categorical variables in the dataset were numerically encoded to facilitate model training. Binary categorical features such as Breastfeeding Status, Bottle Feeding Status, Family Planning Status, Mother Working Status, Food Type, Child Gender, and Ration card were encoded using label encoding, with values assigned as 1 or 2 to represent distinct categories (e.g., yes/no or male/female). Ordinal variables such as Maternal BMI, Mother MUAC, Child Birth Weight, Maternal Education, and Child Age were label-encoded to preserve their rank structure. Nominal categorical variables like Caste and Residence were similarly encoded using integer labels. All remaining features, including anthropometric and demographic measures such as Child Weight, Child Height, Mother Weight, Mother Height, Child MUAC, and Total Child, were treated as either discrete or continuous numeric variables and used without transformation. This encoding strategy ensured compatibility with the modeling pipeline while preserving the interpretability of categorical distinctions.

### 2.4 RISE framework for feature scoring and selection

The traditional machine learning models rely on feature selection driven primarily by model-based importance scores or a single statistical method such as Mutual Information (MI), Chi-Square (Chi^2^), or ANOVA for feature scoring. While these approaches can be effective in identifying statistically significant variables, they often place little to no emphasis on domain-driven feature scoring, which can capture contextually important variables that statistical methods may overlook (25–30).

The proposed methodology introduces a novel approach to feature selection through the RISE framework, which is designed to uncover important features that are underrepresented or overlooked by traditional ML models. Conventional model-based scoring methods tend to favor features with strong statistical patterns, frequently ignoring variables that are contextually significant in real-world scenarios. RISE addresses this imbalance by combining four modules:

(1)  A statistical scoring module—captures relevance based on statistical filter methods
(2)  A frequency boost module—provides the boosting scores based on its top priority
(3)  A domain knowledge scoring module—incorporates expert insight and contextual importance
(4)  A model scoring module—reflects importance as assigned by the machine learning model.

This integrated strategy ensures a more balanced and inclusive selection of features in acute forms of child malnutrition domains.

#### 2.4.1 Statistical scoring module

The statistical scoring module in this study leverages filter-based feature selection techniques to evaluate the importance of features based on their statistical relationship with the target variable. The four key methods used are MI, Chi^2^ test, ANOVA F-test, and ReliefF. Each method has its unique way of assessing relevance, which collectively contributes to a more robust feature evaluation.

##### 2.4.1.1 Mutual information (MI)

Mutual Information measures the dependency between a feature and the target variable. It quantifies how much knowing the value of a feature reduces uncertainty about the target. It captures both linear and non-linear relationships. Features with higher MI scores are considered more informative (26, 28).


$$\mathrm{MI}(X;Y)=\Sigma \;P{\left(x,y\right)}^{*}\;\log(P(x,y)/(P{\left(x\right)}^{*}\;P(y)))$$

##### 2.4.1.2 Chi-square (Chi^2^) test

The Chi-Square test assesses whether there is a significant association between a categorical feature and the target class. It compares the observed frequencies of feature values with the expected frequencies under the assumption of independence. A higher Chi^2^ score indicates a stronger dependency between the feature and the target (28, 29).


$$\chi^{2}=\Sigma ({\left(O\_i-E\_i\right)}^{2}/E\_i)$$

##### 2.4.1.3 ANOVA F-test

ANOVA (Analysis of Variance) is used when the feature is continuous and the target is categorical. It evaluates whether the mean of the feature differs significantly across different classes of the target. A high *F*-value suggests that the feature contributes significantly to class separation (27, 28).


F=MS_B/MS_WwhereMS_B=SS_B/(k1)andMS_W=SS_W/(Nk)

##### 2.4.1.4 ReliefF algorithm

ReliefF is an instance-based feature selection method that considers feature value differences between neighboring instances. It evaluates how well each feature distinguishes between instances of different classes while considering feature interactions and redundancy. It is particularly useful for noisy and complex datasets (30).


$$W[A]=W[A]-{\left(1/m\right)}^{*}\;\Sigma (\text{diff}(A,i,\text{nearHit})-\text{diff}(A,i,\text{nearMiss}))$$

These feature scores, derived before any model training. The selected features based on these scores are then used to train the ensemble boosting model separately for each filter method. The model’s internal feature importance scores are subsequently extracted to evaluate how the model prioritizes features that were pre-selected using different statistical criteria. This approach ensures that the model’s learning is influenced by features deemed relevant purely through statistical assessment, thereby linking filter-based selection with model-driven evaluation.

#### 2.4.2 Frequency boosting module

The Frequency Boosting Module enhances the weight of features that consistently appear as important across multiple independent selection methods. This approach recognizes that a feature is repeatedly identified by statistical filters based ensemble model. Each time a feature is selected or ranked within the top tier by a model, it earns a frequency point. These counts are then normalized and converted into a frequency boosting score, reflecting the stability and consensus around a feature’s importance. By incorporating this module into the RISE framework, features with cross-method agreement are justifiably promoted. This mechanism ensures that consistent signals are amplified, increasing the reliability of the final feature selection.

#### 2.4.3 Domain knowledge scoring module

This Module is designed to integrate expert-driven insights and Contextual relevance into the feature scoring process, leveraging both subject matter expertise and established literature. First, the features were grouped based on thematic relevance, guided by insights from domain experts and collaborators. These groups represent contextual domains that are well-established in influencing the severity of child malnutrition. Next, each feature was assigned an importance score using XGBoost. The scores were then aggregated within their respective groups to calculate a group-wise cumulative score. Based on these cumulative scores, ranks were assigned to each group. Group 1, Child Anthropometry, includes variables such as gender, age, MUAC, weight, and height of the child (29). Group 2 covers Early Feeding Practices, including birth weight, breastfeeding, and bottle feeding (31, 32). Group 3, Maternal Anthropometry, includes the mother’s weight, height, age, BMI, and MUAC (29). Group 4 focuses on Family Structure, including the total number of children and the child’s birth order (33). Group 5 includes Socio-Economic Factors such as residence type, ration card status, caste, and food type. Finally, Group 6 represents Maternal Empowerment, including education, working status, and family planning (34). A numerical rank ranging from 6 (highest relevance) to 1 (lowest) was applied at the group level, and each feature within a group inherited the corresponding rank as presented in Table 1. Finally, domain ranks of all features were normalized between 0–1. These normalized scores serve as the final domain importance values in the RISE framework.

**Table 1:** Domain-based feature grouping and prioritization for acute forms of child malnutrition.

Group priority	Group name	Group feature	Group score	Domain rank
1	Child anthropometry	Child gender, age, MUAC, weight, and height	0.275254	6
2	Early feeding practices	Birth weight, breastfeeding, and bottle-feeding practice	0.246018	5
3	Maternal anthropometry	Mother’s weight, height, age, BMI, and MUAC	0.147003	4
4	Family structure	Total number of children and the child’s birth order	0.127140	3
5	Socio-economic	Residence type, ration card, caste, and food type	0.110287	2
6	Maternal empowerment	Maternal education, working status, and family planning	0.094298	1

Domain-wise grouping and ranking of features influencing acute forms of child malnutrition. Each group consists of thematically related variables aggregated to calculate a Group Score, which indicates the combined influence of that domain. Domain Rank shows the relative priority based on these scores, with higher values indicating greater importance.

#### 2.4.4 A model scoring module

The model scoring module independently generates feature importance scores directly from a boosting model without relying on any prior feature selection or pre-training. In this module, the full set of input features is used to train an ensemble boosting algorithm where the model itself learns which features contribute most to predictive performance.

#### 2.4.5 Final feature scores

The RISE framework computes a final feature score by integrating model-based performance, methodological consistency, and domain relevance. This scoring strategy ensures that selected features are not only powerful predictors but also consistently recognized across methods and grounded in expert knowledge.

The Model Score reflects the feature’s contribution to prediction accuracy, derived directly from a boosting model without any prior filtering. The Frequency boost captures how often a feature is selected as important across a filter-based ensemble model, indicating its robustness and consistency. The Domain Importance represents the contextual and theoretical significance of a feature as determined by thematic grouping guided by subject matter experts.

**Figure 2:**
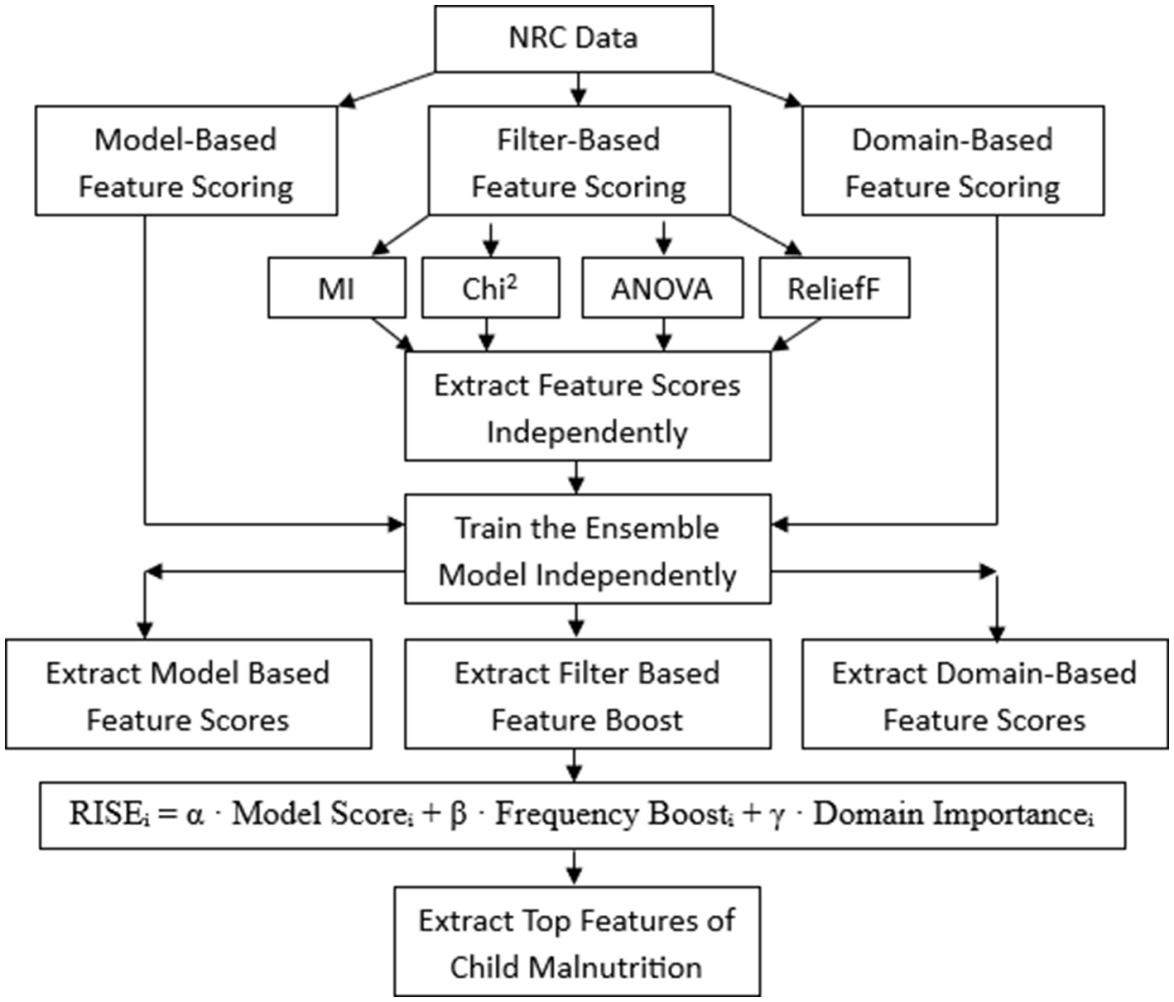
Architecture of RISE framework.

RISE Final Score Formula:


$${\text{RISE}}_{i}=\alpha \cdot {\text{Model Score}}_{i}+\beta \cdot {\text{Domain Importance}}_{i}+\gamma \cdot {\text{Frequency Boost}}_{i}$$

i: Feature index. *α*, *β*, *γ*: Hyper-tuning values to adjust the contribution of each component. In the RISE framework, Selection of Hyperparameter tuning was done using nested validation. The final RISE score is defined as a weighted sum of Model Score, Domain importance, and Frequency Boost. The optimal configuration was determined to be α = β = γ = 0.1. The overall architecture of the RISE framework is presented in Figure 2.

## 3 Results

The dataset used in this study comprises exclusively malnourished children, with the target variable categorically divided into SAM and MAM. The primary objective of this research is to identify the determinants of acute forms of child malnutrition. The analysis focuses on extracting and ranking features through a combination of model-based importance, domain knowledge insights, and frequency-based boosting grounded in a filter-based method. *A priori* power analysis using G*Power (two-tailed, effect size d = 0.5, *α* = 0.05, power = 0.90) indicated that a minimum of 172 participants (86 per group) would be required. This study includes real-time data of 206 children (100 SAM and 106 MAM), ensuring adequate statistical power as well as indicating a balanced distribution between the two groups. The dataset was obtained from the NRC register. All available features were taken for analysis to ensure a comprehensive evaluation and to support the development of a framework for identifying determinants of acute forms of child malnutrition. The study did not use any confounder study, pre-filtering, or exclusion based on correlation thresholds. It retained the full spectrum of variables that may contribute to nutritional outcomes of the child, whether directly or indirectly. This inclusive approach allows the model to assess the relative importance of each feature in context, which ensures completeness and eliminates concerns regarding missing values.

The implementation was done in Python version 3.11.11 (Anaconda distribution). The dataset was first partitioned into training and testing subsets to evaluate generalization performance. Feature selection was guided by XGBoost classifier, statistical importance based on filter-based XGBoost, and domain ranking with XGBoost. To ensure robustness and reduce overfitting, Stratified K-Fold Cross-Validation (with *k* = 5) was employed on the training set. This approach preserved the class distribution across folds and allowed for consistent performance estimation. A nested cross-validation framework was employed for hyperparameter tuning. The outer loop used 5-fold cross-validation to evaluate generalization performance, while the inner loop applied 3-fold cross-validation within a grid search. The optimal values obtained were *α* = *β* = *γ* = 0.1, resulting in a Nested CV mean F1 score of 0.824. For the XGBoost classifier, parameters specified were the number of estimators (n_estimators = 100), evaluation metric (eval_metric = logloss), and random seed (random_state = 42). All other hyperparameters were retained at their default values (learning_rate = 0.1, max_depth = 6, subsample = 1.0).

The XGBoost, a high-performance gradient boosting algorithm, is widely used for its accuracy, speed, and ability to handle complex, structured data (35–38). This section presents the experimental implementation of the RISE framework using the XGBoost model. However, the proposed approach is flexible and can be generalized to other machine learning ensemble methods for enhanced applicability. The results of model-based and domain-based feature scoring are summarized in Table 2. The model-based scores were derived by training an XGBoost classifier independently on a curated set of input features and extracting importance, which reflects each feature’s contribution to child malnutrition. In parallel, domain ranks were obtained based on thematic relevance, guided by insights from domain experts and group score, and it is further normalized to ensure symmetry in the RISE score components, as presented in Table 1.

**Table 2:** Feature importance from XGBoost model and normalized domain scores.

Feature	Model importance	Domain rank (normalized)
Child weight	0.1304	1.0
Child MUAC	0.0989	1.0
Breastfeeding status	0.0969	0.8
Child height	0.0820	1.0
Child order	0.0669	0.4
Mother age	0.0550	0.6
Food type	0.0539	0.2
Mother weight	0.0485	0.6
Child gender	0.0453	1.0
Maternal BMI	0.0443	0.6
Mother height	0.0443	0.6
Mother MUAC	0.0411	0.6
Residence	0.0340	0.2
Maternal education	0.0336	0.1
Child birth weight	0.0279	0.8
Total child	0.0252	0.4
Child age	0.0248	1.0
Bottle feeding status	0.0220	0.8
Caste	0.0139	0.2
Family planning status	0.0112	0.1
Maternal WORKING STATUS	0.0000	0.1
Ration card	0.0000	0.2

Presents XGBoost model importance and normalized domain scores. The domain scores are adapted from domain-derived weightings as shown in Table 1 and further normalized between 0–1.

To identify the most influential factors associated with acute forms of child malnutrition, filter-based feature selection methods were employed. Each method prioritized the top 15 features based on their statistical relevance to the target outcome. These selected features were used independently to train XGBoost models. This approach allowed us to assess how feature selection strategies influence model performance and feature importance scoring. Further, each feature earns its frequency boost score based on the number of times it appears in the top 10 ranked features across four different filter-based feature selection methods. If a feature appears in the top 10 of all four methods, it is assigned a boost value of 4. If it appears in three methods, the boost value is 3; in two methods, it is 2, and in one method, it is 1. If the feature does not appear in the top 10 of any method, the frequency boost is 0. This scoring mechanism reflects the consistency and recurrence of feature importance across multiple selection methods. The results of filter-based XGBoost, along with frequency boost, are presented in Table 3. The performance of the XGBoost model across various feature training configurations, its accuracy, recall, precision, and F1-scores are presented as a unified performance report in Figure 1.

**Table 3:** Consolidated feature importance scores from XGBoost models using multiple filter methods and associated frequency boosting.

SL. no	Feature	MI+XGB	CHI2 + XGB	ANOVA+XGB	ReliefF + XGB	Frequency boost
1	Child weight	0.17623	0.07254	0.07823	0.08677	4
2	Mother weight	0.07228	0.06940	0.06450	0.08097	4
3	Child age	0.05977	0.05965	0.04656	0.07366	4
4	Child order	0.11333	0.08627	0.07579	0.09466	4
5	Mother MUAC	0.03908	0.08464	0.06795	0.10448	3
6	Maternal BMI	0.07584	0.03560	0.04508	0.07273	3
7	Maternal education	0.02548	0.07033	0.10025	0.13661	3
8	Food type	-	0.22474	0.30430	-	2
9	Child MUAC	-	-	0.05691	0.08130	2
10	Child gender	0.09670	0.06540	-	-	2
11	Family planning status	0.06051	-	-	0.05844	2
12	Child birth weight	0.04554	0.05052	0.04102	0.04506	2
13	Residence	-	0.05299	-	-	1
14	Bottle feeding status	0.07320	-	-	-	1
15	Total child	0.01176	-	-	0.06862	1
16	Mother age	0.01176	0.05808	0.02073	-	1
17	Child height	0.13852	-	-	-	1
18	Mother height	-	-	0.03518	0.04935	0
19	Breastfeeding status	-	-	-	0.04736	0
20	Caste	-	0.04637	0.04038	-	0
21	Maternal working status	-	0.02346	0.02313	0.000000	0
22	Ration card	0.000000	0.000000	0.000000	0.000000	0

The table presents consolidated feature importance scores for acute forms of child malnutrition. The importance scores are obtained from XGBoost models trained using various filter methods: MI, CHI2, ANOVA, and ReliefF. Only features that appear in the top 10 of at least one configuration were given frequency boost score. The Frequency Boost Score indicates the feature’s presence across top-10 lists: 4 – feature appears in the top 10 of all 4 methods. 3 – feature appears in the top 10 of any 3 methods. 2 – feature appears in the top 10 of any 2 methods. 1 – feature appears in the top 10 of any 1 method. 0 – feature does not appear in the top 10 of any method.

**Figure 3:**
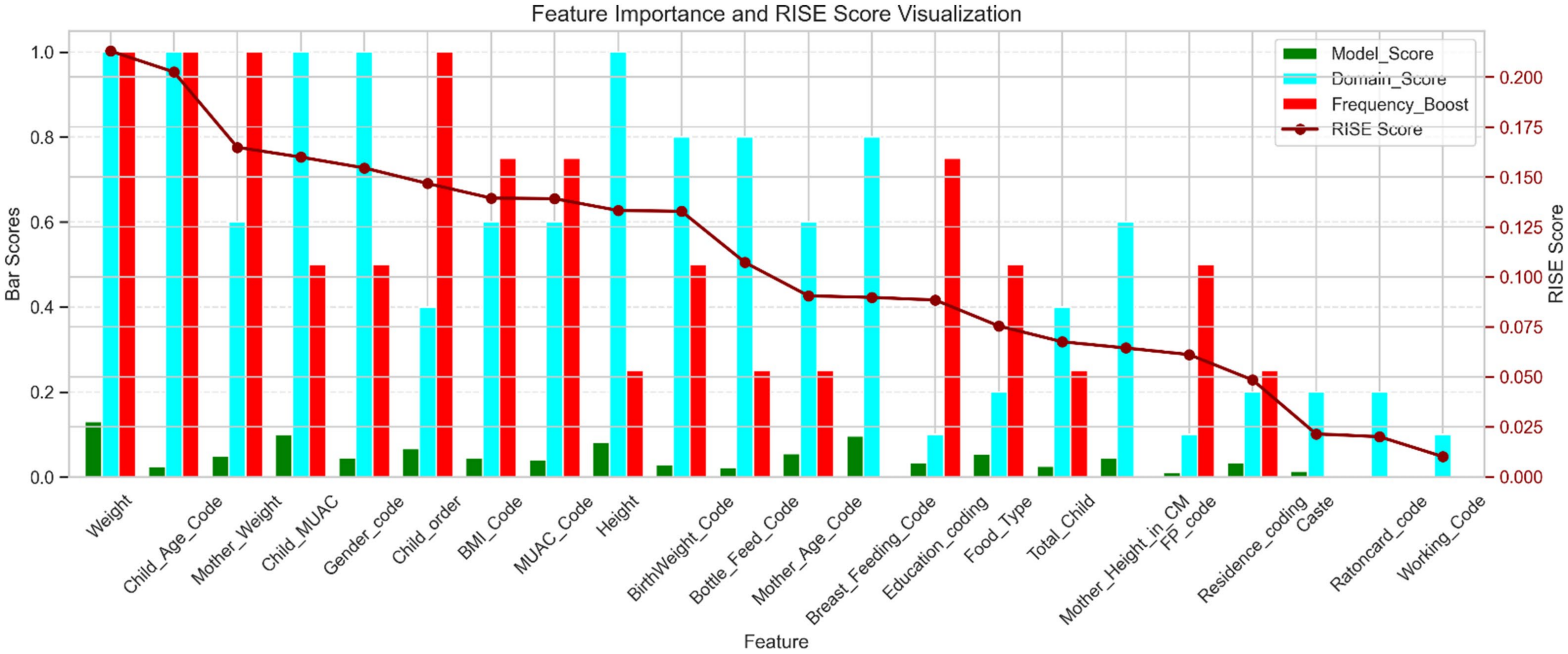
Feature importance and RISE score visualization. This figure presents a comparative analysis of feature importance using four scoring metrics: model score: quantifies each feature’s predictive contribution based on XGBoost. Domain score: reflects relevance based on thematic grouping and domain knowledge. Frequency boost: consistent top 10 features in filter-based XGBoost. RISE score: a composite metric integrating the above three scores to represent overall feature relevance.

Now, the RISE framework is employed by integrating three key components: domain importance scoring, model-based feature scoring, and frequency-based boosting. Each feature’s final score is computed by combining these elements using a weighted formula, enabling a balanced representation of statistical relevance, expert knowledge, and selection consistency presented in Table 4. This comprehensive scoring strategy ensures that both high-performing and domain-relevant but underrepresented features are prioritized appropriately in the final analysis, visualized in Figure 3.

**Table 4:** Final RISE with normalized domain score, frequency boost, and domain score with corresponding hyper tuning values.

Feature	Domain score	Model score	Frequency boost	RISE score
Child weight	1.0	0.1304	1.00	0.2130
Child age	1.0	0.0248	1.00	0.2025
Mother weight	0.6	0.0485	1.00	0.1648
Child MUAC	1.0	0.0989	0.50	0.1599
Child gender	1.0	0.0453	0.50	0.1545
Child order	0.4	0.0669	1.00	0.1467
Maternal BMI	0.6	0.0443	0.75	0.1394
Mother MUAC	0.6	0.0411	0.75	0.1391
Child height	1.0	0.0820	0.25	0.1332
Child birth weight	0.8	0.0279	0.50	0.1328
Bottle feeding status	0.8	0.0220	0.25	0.1072
Mother age	0.6	0.0550	0.25	0.0905
Breastfeeding status	0.8	0.0969	0.00	0.0897
Maternal education	0.1	0.0336	0.75	0.0884
Food type	0.2	0.0539	0.50	0.0754
Total child	0.4	0.0252	0.25	0.0675
Mother height	0.6	0.0443	0.00	0.0644
Family planning status	0.1	0.0112	0.50	0.0611
Residence	0.2	0.0340	0.25	0.0484
Caste	0.2	0.0139	0.00	0.0214
Ration card	0.2	0.0000	0.00	0.0200
Working code	0.1	0.0000	0.00	0.0100

The table presents consolidated feature scores for malnutrition-related data. The RISE_Score represents the final integrated score for each feature, calculated by combining: 1. Domain score – provided based on thematic grouping and expert ranking and further normalized between 0–1. 2. Model score – provides the feature importance obtained from the XGBoost classifier. 3. Frequency boost – provided for the feature that is repeatedly identified by statistical filters based ensemble model. The combination is controlled by hyperparameters α, β, and γ, which were optimized using nested cross-validation (nested CV). The optimal values obtained were α = β = γ = 0.1, resulting in a nested CV mean F1 score of 0.824.

## 4 Discussion

**Figure 4:**
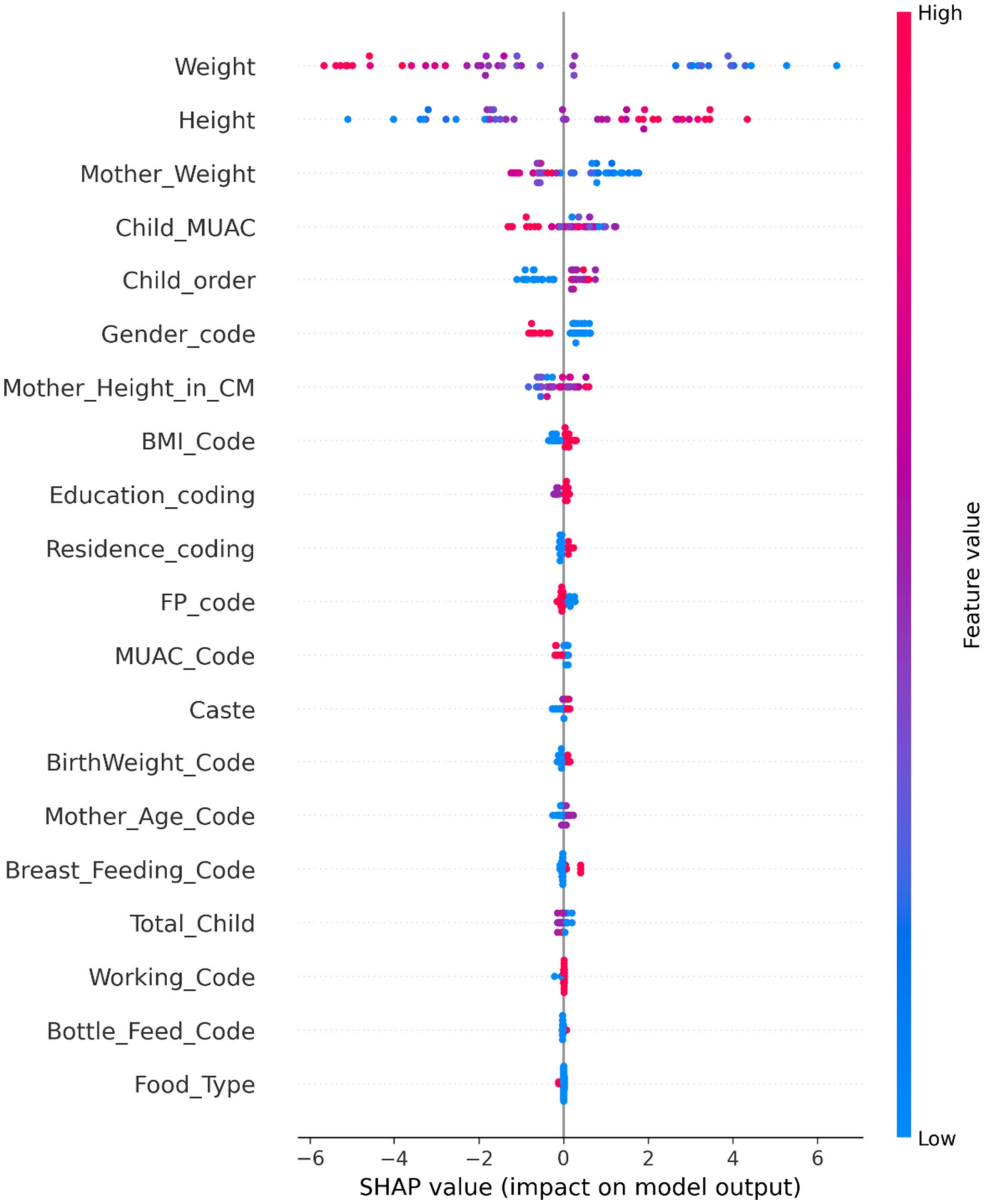
SHAP summary plot showing feature contributions for acute forms of child malnutrition.

The analysis of top-ranked features using the RISE framework reveals critical insights into the multifaceted nature of acute forms of child malnutrition. Among the various determinants, child anthropometric indicators emerged as the most influential, followed by maternal anthropometry and child order. However, Data from the NRC reinforces these findings: over 88% of malnourished children were being breastfed, including 58.51% of MAM cases and 44.32% of SAM cases. Furthermore, 49.0% of mothers of children diagnosed with SAM had an inadequate BMI, indicating undernutrition. Similarly, a higher proportion, 56.6% of mothers of children with MAM, also exhibited inadequate BMI levels (8). This strongly suggests an interlinked pattern that mothers with poor nutritional status may be breastfeeding children who consequently face an increased risk of malnutrition, emphasizing the dual burden of maternal and child undernutrition. The study also implements SHAP (SHapley Additive Explanations), which indicates how each feature contributes to the model’s predictions. The SHAP results further validate that maternal anthropometry, such as the mother’s weight, plays a significant role in acute forms of child malnutrition presented in Figure 4. Child order reflects the burden of familial resource allocation, with 56.31% of malnourished children being of second birth order or higher, indicating that increasing family size may dilute maternal attention and care (9, 10), further confirmed with SHAP analysis presented in Figure 4. Together, the integrated model insights and contextual statistics highlight critical intervention points. The evidence highlights the importance of policy strategies that enhance maternal nutrition, target vulnerable groups, and support young mothers, particularly those breastfeeding, in effectively breaking the intergenerational cycle of malnutrition (29, 30).

This research offers a novel contribution to the field of Child malnutrition and Machine learning. Firstly, in the child malnutrition domain, multi-dimensional and data-driven exploration of the complex determinants of acute forms of child undernutrition is achieved through integrated analysis of Maternal and Child Factors, emphasizing the mother–child nutritional dyad as a central axis of malnutrition (29). This integrated approach moves beyond child-focused indicators and reflects a life-cycle and intergenerational perspective on malnutrition. Secondly, this research introduces a novel and comprehensive feature prioritization framework, RISE (Relevance-based Integration of Statistics and Expertise), that represents a significant advancement in the field of child malnutrition analytics. Unlike conventional studies that depend on machine learning feature importance or subjective domain prioritization (7–17), this study uniquely integrates multi-dimensional evidence by combining four robust statistical filter methods (Mutual Information, Chi-square, ANOVA-F, ReliefF) with an ensemble model, domain-based scoring, and a newly proposed frequency boosting technique that captures the cross-method consensus of feature relevance. This multi-branch integration ensures that underrepresented yet contextually vital variables are not bypassed by purely statistical weightages (27). The RISE framework thus reflects a paradigm shift from purely model-driven selection to an interpretable, explainable, and policy-relevant decision layer. It is an adaptable scoring logic and modular design, suitable for extension to other health and development research areas, establishing both scientific novelty and practical utility.

Some features, such as Mother Height, Breastfeeding Status, Caste, Maternal Working Status, and Ration Card, obtained a frequency boost score of zero. This indicates that none of the statistical feature selection methods consistently identified these variables as important predictors. The likely reasons include weak or indirect associations with acute forms of malnutrition and underrepresentation of certain subgroups in the dataset. Nevertheless, these features remain highly relevant from a public health and domain perspective, as they capture socioeconomic and behavioral dimensions of child nutrition that purely statistical methods may overlook. Therefore, the RISE framework incorporates domain-based correction to ensure such features are not disregarded solely based on low statistical detectability. Limitations of this study include the potential lack of generalizability due to the region-specific and institutionalized nature of the dataset. Such data may reflect only children accessing a particular facility, thereby excluding those without access to the facility or in different regional contexts. Additionally, while the RISE framework offers an advantage by incorporating domain knowledge along with statistical and model-driven methods, it introduces a level of subjectivity, particularly in assigning domain-based weights. Expert input for feature grouping and ranking was not derived through a formal elicitation process. External and temporal validation are essential to ensure the model’s reliability across different populations, settings, and time periods. In the study, the model demonstrates strong performance within the current dataset; its generalizability remains untested.

## 5 Conclusion

By examining the top features ranked by the RISE Score, Child anthropometry emerged as the most influential, followed by maternal anthropometry and child order. This hierarchy underscores a double burden of malnutrition. This reflects the importance of physical growth parameters in assessing nutritional status. Further down, features like maternal anthropometry and child order underscore its influence on child health outcomes. The presence of these variables at the top reinforces the understanding that the importance of maternal nutritional status and physical attributes plays a key role in shaping a child’s growth and development, further highlighting the multifactorial nature of malnutrition.

The RISE framework demonstrates its strength by effectively identifying and assigning importance to features that were under-prioritized by conventional filter-based or model-based feature selection methods. For instance, features such as Ration card = 0.0200, Working status = 0.0100, Caste = 0.0100, Mother height = 0.0644, and Breastfeeding status = 0.0897 lacked frequency boost contributions but were still given significant weight by the RISE framework. This reflects the inclusive nature of RISE, which integrates domain relevance, statistical contribution, and contextual importance, allowing it to highlight features that might be overlooked by purely data-driven models but are crucial in real-world health and nutritional contexts.

Addressing child malnutrition is a crucial step toward achieving Sustainable Development Goals, SDG 2 (Zero Hunger) and SDG 3 (Good Health and Well-being). The development and implementation of the RISE framework is an advancement in malnutrition analytics by bridging the gap between data-driven methodologies and domain expertise. Unlike traditional feature selection methods that may overlook critical variables due to statistical bias, RISE facilitates a more equitable, context-aware, and interpretable feature evaluation. By elevating the significance of factors like maternal education, breastfeeding practices, and socioeconomic conditions, the RISE approach ensures that key public health insights are retained and highlighted in machine learning models. Finally, this framework strengthens the translation of empirical data into actionable knowledge, empowering targeted interventions and evidence-based policymaking in the process of child malnutrition, thus contributing directly to global health equity and the realization of the SDGs.

### 5.1 Future work

While the current model demonstrates strong performance within the study dataset, to ensure reliability, broader applicability, or generalizability, future research should have a strong focus on external and temporal validation. Validating the framework on independent datasets from different geographic regions and across varied time periods, such validation would help in identifying the key determinants of acute child malnutrition.

## Data Availability

The original contributions presented in the study are included in the article/Supplementary material, further inquiries can be directed to the corresponding author. The code is available at: https://github.com/Shruthi-S-Scholar/ChildMalnutrition-rise-framework.
